# The Composition of Expired Air and Its Effects upon Animal Life

**Published:** 1897-03

**Authors:** 


**The Composition of Expired Air and its Effects upon Animal Life.** By J. S. Billings, M.D., S. Weir Mitchell, M.D., and D. H. Bergey, M.D. Pp. 81. Washington: The Smithsonian Institution. 1895.

For many years investigators have been trying to discover the causes of the bad effects of defective ventilation; and whilst some have attributed them to excess of carbon dioxide or deficiency of oxygen, or to both of these factors, there have been others, *e.g.* d'Arsonval and Brown-Séquard, who suppose that even healthy animals exhale a volatile organic poison of the nature of an alkaloid or ptomaine.

Drs. Billings, Weir Mitchell, and Bergey have, in an extensive series of experiments, failed to find any trace of a volatile poison in expired breath. They do find a very small amount of ammonia and combined nitrogen in human breath, and show that this is due to decomposition of organic matter in the mouth and pharynx, but of any peculiar volatile organic poison they find no evidence whatever. They remark that the high mortality of persons living in crowded dwellings is now known to be due in large part to specific diseases, such as phthisis and pneumonia, and that the good effects of increased ventilation are to be attributed in large part to the lessened quantity of dust particles to which micro-organisms adhere. The offensive smell of public halls and places of entertainment is, in their opinion, due to volatile products of decayed teeth and foul mouths, to fatty acids given off in the excretions of the skin, and to the means used for illumination.

This work is issued in large quarto, in the attractive form which characterises all the "Smithsonian Contributions to Knowledge."

